# Isogroup Selection to Optimize Biocontrol Increases Cannibalism in Omnivorous (Zoophytophagous) Bugs

**DOI:** 10.3390/insects8030074

**Published:** 2017-07-25

**Authors:** François Dumont, Denis Réale, Eric Lucas

**Affiliations:** Département des Sciences Biologiques, Université du Québec à Montréal, CP 8888, Succ. Centre Ville, Montréal, QC H3C 3P8, Canada; reale.denis@uqam.ca (D.R.), lucas.eric@uqam.ca (E.L.)

**Keywords:** cannibalism, genetic diet specialization, zoophytophagous predators, isogroup lines, plant bugs, artificial selection, biological control, *Campylomma verbasci*

## Abstract

Zoophytophagous insects can substitute animals for plant resources when prey is scarce. Many arthropods feed on conspecifics to survive in these conditions. An individual’s tendency for cannibalism may depend on its genotype along with its diet specialization, in interaction with the availability of alternative food resources. We compared two isogroup lines of the zoophytophagous mullein bug, either specialized on animal or on plant diets, that were generated to improve biocontrol. We predicted that: (1) bugs from the prey-specialized line would show higher levels of cannibalism than bugs from the pollen-specialized line, and (2) both lines would decrease cannibalism levels in the presence of their preferred resource. Under laboratory conditions, large nymphal instars had 24 hours to feed on smaller instars, in the absence of additional resources, or with either spider mites or pollen present. Cannibalism was reduced by the availability of both prey and pollen, although prey had a lower effect than pollen. The intensity of cannibalism was always higher in the prey-specialized line than in the pollen-specialized line, regardless of the availability of supplemented resources. The pollen-specialized line had decreased cannibalism levels only when pollen was available. These results indicate that cannibalism is a potentially regulating force in the prey-specialized line, but not in the pollen-specialized line.

## 1. Introduction

Cannibalism (i.e., intraspecific predation) is common among natural populations of insects and other arthropods [1,2]. In her review, Fox [1] reported that cannibalism is frequent in omnivorous predators, which can feed on several alternative food resources. Cannibalism results from opportunistic predation on vulnerable individuals or stages, and should increase with the population density. Moreover, feeding on conspecifics may allow predatory organisms to complete their development under conditions of low-quality resources [3,4,5].

Resource availability can modulate cannibalism in zoophytophagous predators (i.e., omnivorous predators that can fully substitute zoophagy for phyophagy) [6,7,8,9]. Leon-Beck et al. [6] observed that increased prey and pollen availability decreased cannibalism levels equally in the zoophytophagous minute pirate bug *Orius laevigatus* (Say; Hemiptera: Anthocoridae). Similarly, the twelve-spotted ladybug beetle *Coleomegilla maculata* (De Geer; Coleoptera: Coccinellidae) has decreased cannibalism levels when pollen is available [9]. However, individuals or genotypes specialized on particular types of a resource may show different levels of cannibalism as a result of changes in the food resource. In recent studies [10], we found genetic specialization for prey or pollen diets in the mullein bug *Campylomma verbasci* (Meyer; Hemiptera: Miridae). In the presence of pollen, pollen-specialized genotypes switched from feeding on prey to feeding on pollen, whereas more zoophagous genotypes continued to feed on prey. Moreover, we previously reported a positive genetic correlation between the level of zoophagy on a primary prey (spider mites) and on a secondary prey (aphids) in the mullein bug [11]. Thus, individuals (or a genotype) specialized on prey resources could overcome prey scarcity by feeding on conspecifics (animal resources), whereas others instead feed on plant resources.

In this study, we used two isogroup lines contrasting in their levels of zoophagy and diet specialization to assess whether, depending on resource availability, genetic differences in diet specialization are linked to the intensity of cannibalism. These isogroup lines were developed in order to improve the biocontrol of orchard pests. We predicted that: (1) the isogroup line specialized on prey would show higher levels of cannibalism than the pollen-specialized line, and (2) both lines would increase their cannibalism levels in response to the absence of their specialized resource.

## 2. Methods

### 2.1. Populations and Rearing Conditions

In previous studies, we determined the level of zoophagy [11] and diet specialization [10] of 12 isogroup lines. Here, we used two isogroup lines that exhibited contrasting levels of zoophagy on spider mites. Both lines were founded from two virgin females and two males, either captured as nymphs on apple trees or the mullein plant in apple orchards in different regions of Québec (Canada) in 2011 and 2012, or from a stock population based on individuals captured during the previous years. Details on the foundation and breeding of lines are described by Dumont et al., and can be found in [10,11]. Individuals within the isogroup lines were allowed to reproduce for 15 generations (assuming a generation every 40 days). This number of generations was high enough to allow within-line genetic drift to be reduced, relative to the among-line genetic variability [11]. In the present study, we compare the rates of cannibalism (i.e., number of intraspecific prey killed per 24 h) of older nymphal stages (i.e., N4 and N5) from the two lines on younger nymphal stages (i.e., intra-specific prey N1 and N2). N1 and N2 nymphs were randomly picked from the stock population to avoid uncontrolled interference of prey defensive behavior on the rate of cannibalism of each of the two lines.

### 2.2. Cannibalism Tests

Prior to the beginning of the tests, the older nymphs were individually placed for 24 h in a 10 cm diameter Petri dish containing a cutting of mullein leaf in agar gel. Cannibalism tests were run in 5 cm diameter Petri dishes containing a fresh cut of bean leaf embedded upside down in agar gel. The Petri dishes had a hole in the lid covered with fine muslin for humidity control. The bean leaf covered all the space in the Petri dish, thus providing a surface area of 19.64 cm^2^ to the nymphs. In addition to the control (i.e., no resource treatment), two treatments were provided: a pollen treatment (a small quantity of pollen, about 0.5 g, was deposited on the middle of the Petri dish) and a prey treatment (i.e., a 1 cm^2^ cutting of bean leaf well-infested with two-spotted spider mites was deposited in the middle of the Petri dish). Just before the beginning of the test, four young nymphs (N1 and/or N2) were gently introduced into the Petri dish with a small paintbrush. Two older nymphs (N4 and/or N5) were then introduced into the Petri dish. The Petri dishes were then closed with parafilm and placed into a growth chamber at 25 °C, 70% relative humidity., and 16:8 hours Light:Dark for 24 h. The rate of cannibalism was measured from the number of nymphs killed after this period. Mullein bugs consume their prey and empty their carcasses, and thus cannibalism of mullein bugs can easily be differentiated from natural death. The rate of cannibalism (the dependent variable) of each line was tested 20 times in each of the three treatments (i.e., control, spider mites or pollen) for a total of 120 individuals tested (*n* = 120).

### 2.3. Statistical Analysis

First, a generalized linear model (GLM) with a Poisson distribution and a log link function was implemented to test for the difference among the treatments (i.e., control, prey and pollen treatments), the type of diet specialization (prey- or pollen-specialized line), and their two-way interaction. Variables were selected using a backward stepwise procedure, and only variables with a *p*-value under 0.05 were retained in the models. The *p*-value of each variable was obtained using the *drop1* function for the Poisson GLM, which drops each explanatory variable in turn, and compares differences among models to a Chi-square distribution [12]. An all-pairwise comparison of Tukey’s test was implemented using the *glht* function (package *multcomp* [13]) to detect differences among the three environmental conditions [14,15]. Then, two GLM models for the Poisson-distributed response variable were separately run to test the effect of the treatment on the cannibalism of each line. All analyses were performed using R [16].

## 3. Results

**Figure 1:**
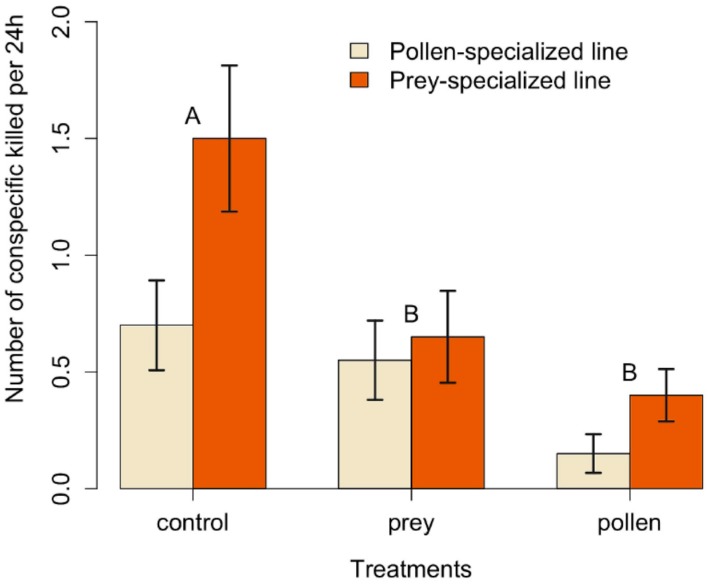
Rate of cannibalism (number of intraspecific prey killed per 24 h) in mullein bugs of prey- and pollen-specialized lines fed on diets without additional food (control) or with two-spotted spider mites and pollen. Letters correspond to significant differences among treatments (α = 0.05).

Mullein bugs killed 1.10 ± 1.22 (mean ± SD) nymphs per day in the control group, 0.76 ± 0.81 in the prey treatment, and 0.28 ± 0.45 in the pollen treatment, respectively (Figure 1). Differences among treatments were statistically significant (LRT (likelihood ratio test) = 21.52; *p* < 0.0001), indicating that both the prey and pollen treatments reduced cannibalism (Table 1).

The rate of cannibalism was significantly higher in the prey-specialized line (0.85 ± 1.09 nymphs per day) than in the pollen-specialized line (0.47 ± 0.72 nymphs per day), regardless of the treatment provided (LRT = 6.79; *p* = 0.009; Figure 1).

The two-way interaction between the treatment and the line was not statistically significant, and was thus removed from the model (LRT = 1.68; *p* = 0.43). However, the availability of both prey and pollen significantly decreased cannibalism levels in the prey-specialized line (Table 2), whereas only pollen effectively decreased cannibalism levels in the pollen-specialized line (Table 2).

## 4. Discussion

Cannibalism can be an important source of mortality under both laboratory and field conditions [1,2,17]. In zoophytophagous predators, the availability of alternative prey or plant resources is assumed to reduce the occurrence of cannibalism [6]. However, diet specialization can modulate how individuals or genotypes interact with alternative food resources [11,18,19], including conspecifics [20]. Our study shows that mullein bug isogroup lines that differ in their levels of zoophagy and diet specialization had significant differences in the levels of cannibalism. The prey-specialized line exhibited higher levels of cannibalism than the pollen-specialized line, regardless of whether or not alternative resources (i.e., pollen or prey) were available, a result that supports our first prediction. However, contrary to our second prediction, the prey-specialized line decreased its cannibalism levels in response to the presence of both prey and pollen. Nonetheless, the response to resource availability was different between the two lines; the pollen-specialized line only had decreased cannibalism levels when the preferred resource (i.e., the pollen) was available.

In addition to its effect on the population density (through an increased mortality), cannibalism can also affect both population dynamics and population structure, as it is often asymmetric (i.e., larger stages feed on smaller vulnerable stages) [2,8,21,22,23,24]. Population density, structure and dynamics are critical ecological concepts in biological control and crop protection [8,21,22]. These ecological concepts are especially important when it comes to zoophytophagous predators, and can either be beneficial when they feed on prey, or harmful when they feed on plant materials [25]. For instance, nymphs of mullein bugs provide benefits in the agroecosystem when feeding on spider mites or aphids [26], but can be harmful when feeding on apple fruitlets [27,28]. Damages caused to crops by zoophytophagous species usually increase when their population is high relative to their prey populations [29,30]. Thus, cannibalism should have the desirable effects (in terms of crop protection) of decreasing the population density of zoophytophagous predators in crop systems when pests are less abundant [23].

Given that conspecifics can serve as a surrogate for prey during periods of prey scarcity, and that cannibalism can regulate a zoophytophagous predator’s population [6], a potential increase in cannibalism in the absence of prey would be beneficial to crop protection. However, we observed that protein-rich plant resources (i.e., pollen) strongly prevent cannibalism in zoophytophagous mullein bugs. The consequences of the absence of prey would be important; the mullein bug population should remain relatively high and close to the flower (or newly formed fruitlets), where the bugs cause significant damages to crop [28]. Thus, as long as pollen is available to nymphs, cannibalism would be only a marginal factor affecting the mullein bug density. However, pollen is usually not available to the late nymphal stages (e.g., fourth and fifth stage) of the first generation of mullein bugs, which grow on apple trees [31] (Bartlett 1996). These stages would have the choice to feed on developing fruitlets, a low-quality resource [32], or on conspecifics when prey is not available. Feeding on conspecifics rather than fruitlets would have considerable consequences for the level of damage on apple fruit caused by mullein bug nymphs.

The differences in cannibalism between the prey- and pollen-specialized lines in zoophytophagous mullein bugs suggest an additional interest for using more zoophagous lines in the agroecosystem. The prey-specialized line potentially provided higher benefits for crop protection by preferentially choosing prey over plant resources [11] and exhibiting a high level of zoophagy [10]. Additionally, a high propensity for cannibalism when main resources are unavailable (i.e., spider mites and pollen) would decrease the mullein bug population density, and divert nymphs from feeding on apple fruitlets during a risk period of damages to crops by mullein bugs. For instance, Pels and Sabelis [33] observed that the predatory mite *Phytoseiulus persimilis* Athias-Henriot (Acari: Phytoseiidae) delayed their dispersal from the feeding patch when they had cannibalism opportunities. Mullein bug nymphs that preferentially feed on prey should be found on apple leaves rather than on the flower, and their movement to the flower could be delayed when they can prey on conspecifics. Thus, applying artificial selection to a zoophytophagous predator population favoring preferences for prey and with high levels of aggressiveness could theoretically increase the benefits (increase zoophagy on agricultural pests), while decreasing the risk associated with their presence on crops (by increasing cannibalism during high-risk periods of damages to crops). The manipulation of the composition of mullein bug populations through artificial selection could be a means to take advantage of this predator in apple orchards [34,35].

## 5. Conclusions

Isogroup selection aiming to increase economically relevant traits (i.e., level of zoophagy) in zoophytophagous predators can have consequences on other traits or behaviors. In our study, highly zoophagous and prey-specialized isogroup lines were more cannibalistic than lowly zoophagous and pollen-specialized lines, no matter the type of food resources available. Therefore, manipulating the genetic composition of zoophytophagous predators could alter their ecological interactions (including interactions with their conspecifics).

## Figures and Tables

**Table 1:** All-pairwise comparisons of Tukey’s Test for Poisson GLM-compared treatments (control: no added food; prey: two-spotted spider mites; and pollen) in tests of cannibalism in mullein bug nymphs (both prey- and pollen-specialized lines were included in the model).

Comparisons	Estimates ± s.e.	z-Value	*p*-Value
Prey–Control	−0.61 ± 0.25	−2.39	0.04
Pollen–Control	−1.39 ± 0.34	−4.11	<0.001
Prey–Pollen	0.78 ± 0.36	2.14	0.08

**Table 2:** The effect of two-spotted spider mites and pollen availability on the rate of cannibalism (intraspecific prey per 24 h) in prey- and pollen-specialized lines of mullein bugs (lines were tested separately).

Treatment	Estimates (± SD)	z-Value	*p*-Value
Prey-Specialized Line			
Pollen	−1.32 ± 0.40	−3.32	0.0009
Prey	−0.84 ± 0.33	−2.52	0.01
Pollen-Specialized Line			
Pollen	−1.54 ± 0.64	−2.42	0.02
Prey	−0.24 ± 0.40	−0.60	0.55
